# Partnerships to Connect Aging Research to Policy

**DOI:** 10.1093/geroni/igaa057.2386

**Published:** 2020-12-16

**Authors:** Kali Thomas

**Affiliations:** Brown University, Providence, Rhode Island, United States

## Abstract

The fifth speaker is Dr. Kali Thomas. Dr. Thomas will discuss her experience working in partnership with government and non-profit organizational stakeholders to conduct aging research that informs policy and practice. Dr. Thomas is an Associate Professor at the Brown School of Public Health and research health science specialist at the Providence VA Medical Center, where her research focuses on identifying ways to improve the quality of life of older adults needing long-term services and supports (LTSS) through applied health services research.

